# Effects of Acute Hypoxia on Early Visual and Auditory Evoked Potentials

**DOI:** 10.3389/fnins.2022.846001

**Published:** 2022-04-25

**Authors:** Kara J. Blacker, Daniel G. McHail

**Affiliations:** Naval Medical Research Unit-Dayton, Wright-Patterson Air Force Base (AFB), Dayton, OH, United States

**Keywords:** hypoxia, evoked potentials, P100, sensory gating, human performance

## Abstract

Reduced levels of environmental oxygen lead to hypoxic hypoxia and are a primary threat in tactical aviation. The visual system is particularly vulnerable to hypoxia, and its impairment can severely impact performance. The auditory system is relatively spared by hypoxia, although which stages of auditory processing are most impacted by hypoxia remains unclear. Previous work has used electroencephalography (EEG) to assess neural markers of cognitive processing for visual and auditory stimuli and found that these markers were sensitive to a normobaric hypoxic exposure. In the current study, we assessed whether early sensory evoked potentials, that precede cognitive activity, are also impaired by normobaric hypoxia. In a within-subjects design, we compared visual (P100) and auditory evoked potentials (sensory gating for the P50, N100, and P200) in 34 healthy adults during normoxic (21% O2) and two separate hypoxic (9.7% O_2_) exposures. Self-reported symptoms of hypoxia were also assessed using the Hypoxia Symptom Questionnaire (HSQ). We found that P100 mean amplitude was not reduced under hypoxic compared to normoxic conditions, suggesting no statistically significant impairment of early visual processing. The sensory gating ratio for auditory stimuli was intact for paired responses of the P50 and N100. However, the P200 sensory gating ratio was attenuated under hypoxic compared to normoxic conditions, suggesting disruption of the auditory system specific to the level of allocating attention that follows basic auditory processing. Exploratory analyses of HSQ scores identified a robust effect of hypoxia. However, consistency of symptoms reported between the two hypoxia exposures exhibited high intra-individual variability, which may have implications for the theory that individuals have a consistent hypoxia signature or reliable constellation of responses to hypoxia. These findings suggest that early sensory processing is not impaired during hypoxia, but for the auditory system there is impairment at the level of attentional processing. Given the previous findings of impaired visual performance under hypoxia, these results suggest that this impairment does not stem from early visual processing deficits in visual cortex. Together these findings help focus the search on when and where hypoxia-induced deficits occur and may guide the development of countermeasures for hypoxia in tactical aviation.

## Introduction

Exposure to reduced levels of breathable oxygen results in hypoxic hypoxia. Healthy individuals may encounter hypoxic conditions at high altitudes that occur during aviation or mountain climbing. Hypoxic exposure in healthy individuals is known to impair a number of perceptual and cognitive processes (e.g., Fowler et al., 1987; Temme et al., 2010; Malle et al., 2013). The impact of hypoxia on a variety of performance outcomes has received much attention in recent years within the military aviation community due to the threat of hypoxia in tactical aircraft (Elliott and Schmitt, 2019).

The negative impact of hypoxia exposure on vision is well-established in the literature and has implications for a number of human performance outcomes. The visual system is sensitive to its oxygen supply at multiple levels including the retina, photoreceptors, and cortical and sub-cortical pathways. Estimates of visual system projections throughout the cortex are thought to be in the hundreds, with interactions beyond visual cortex into areas such as frontal, temporal, parietal lobes, and the midbrain (Mather, 2016). Thus, cortical insults either from injury or hypoxia are more likely to lead to a visual perception deficit (Barbur and Connolly, 2011; Greenwald et al., 2012). Additionally, unique metabolic requirements for the visual system result in consumption both in light and darkness. Cone photoreceptors, which enable color vision, consume more energy than rods, as rods do not saturate in bright light. In total, visual processing ranks as one of the highest energy- and oxygen-demanding systems of the brain (Wong-Riley, 2010). Due to these demands, it is unsurprising that exposure to hypoxic conditions results in changes to the perception of light intensity (Fowler et al., 1993) and impairs color vision (Connolly et al., 2008; Barbur and Connolly, 2011). Additionally, some of the most commonly reported symptoms of hypoxia include graying, tunnel vision, and blurry vision (Woodrow et al., 2011).

While the effects of hypoxia on the visual system are profound and consistent in the literature, the effects on the auditory system are less straightforward. For example, basic auditory sensitivity, as measured with pure-tone audiometry, appears to be relatively unaffected by low-oxygen exposure (Burkett and Perrin, 1976; Watson et al., 2000; but also see, McAnally et al., 2003; Lucertini et al., 2020). However, other aspects of auditory processing may be affected by hypoxia such as vulnerability to noise (Fehrenbacher et al., 2021) or sound localization (Rosenberg and Pollard, 1992). These other aspects of auditory performance, like sound localization are especially relevant in an aviation environment when considering how hypoxia may affect in-cockpit spatial audio cueing systems.

Neurophysiological effects of hypoxia on early processing in the auditory system have also been assessed using electroencephalography (EEG) across a variety of hypoxia exposure conditions. Auditory brain-stem responses (ABRs) occur within 10 ms after stimulus presentation, and studies have found that acute hypobaric hypoxia impaired ABRs at 17,000 ft (Urbani and Lucertini, 1994) and 14,750 ft (Hayashi et al., 2005). In addition, the latency of auditory steady-state responses has been found to increase after consecutive hypobaric hypoxia exposures at a simulated altitude of 17,000 ft (Lucertini et al., 2002). Middle latency auditory evoked potentials occurring between approximately 10–80 ms after stimulus presentation, in contrast, may be relatively less sensitive to hypoxia (Lucertini et al., 1993; Bouchet et al., 1997). Similarly, Hayashi et al. (2005) found that N100 amplitude and latency were not affected by a hypobaric hypoxia exposure at 14,750 ft.

Previous work has demonstrated that hypoxic exposure disrupts the mismatch negativity (MMN) and P3a event-related potentials (ERP; Seech et al., 2020; Blacker and McHail, 2021; Blacker et al., 2021). The MMN/P3a complex is a reliable neural index of automatic and preattentive stages of information processing. Previous work has demonstrated that these ERPs are disrupted both within the auditory (Seech et al., 2020; Blacker and McHail, 2021) and visual (Blacker et al., 2021) domains. Broadly speaking, ERPs are often classified as “cognitive” or “sensory” ERPs. Later ERPs in approximately the 100–400 ms range reflect cognitive processing of external stimuli and tend to involve attention, working memory, decision-making, etc. This cognitive processing of sensory stimuli is preceded by stimulus transduction in sensory organs and conduction of neural signals along the sensory pathways (Pratt, 2011). Thus, the previous work showing that the MMN and P3a components are disrupted during hypoxia fall into the category of cognitive ERPs. However, it seems plausible that decreased levels of breathable oxygen might impact sensory inputs prior to the subsequent cognitive processing of those stimuli. Therefore, in the current study we sought to examine early sensory evoked potentials to test whether early visual and auditory processing that precedes cognitive activity is also disrupted during hypoxia.

Specifically, here we were interested in early visual and auditory evoked potentials. Visual evoked potentials, such as the P100, are the electric manifestation of cortical and subcortical activation of the visual pathway (Celesia and Brigell, 1999). In order to elicit a P100, the typical stimulus used is a black and white checkerboard pattern that reverses at a set rate without any changes in total luminance. This type of stimuli is often used in clinical assessments of the visual pathway because it provides small inter-individual and intra-individual test-to-test variability and high sensitivity to impaired conduction along the visual pathways (Pratt, 2011). For example, the visual P100 is delayed in latency in patients with multiple sclerosis, which is caused by persistent demyelination that slows nerve conduction in the central nervous system (McDonald and Halliday, 1977; Niklas et al., 2008). To the best of our knowledge, the visual P100 has not been tested under hypoxic conditions.

While previous work, discussed above, has examined the effects of hypoxia on ABRs and middle-latency auditory potentials, here we wanted to examine long-latency auditory potentials, specifically those that index sensory gating. Long latency potentials include an initial positive peak around 50 ms (P50), followed by a large negative waveform (N100), and finally a positive going potential around 200 ms (P200). While the P50 and N100 are linked to sensory processing, later waveforms like the P200 are considered to reflect processing beyond sensory perception (Pratt, 2011). This series of auditory potentials can be used to measure an adaptive neural function known as sensory gating. Sensory gating represents the nervous system’s ability to inhibit responding to irrelevant environmental stimuli (Pratt, 2011). This is thought to be a protective mechanism for subsequent cognitive processing (Lijffijt et al., 2009). P50, N100, and P200 sensory gating can be measured with a paired-clicks paradigm (Wood and Wolpaw, 1982). In this paradigm, a set of simple auditory tones presented in close proximity elicits the auditory potentials of interest. The amplitude is attenuated for the second stimulus (S2) compared to the first stimulus (S1) in the pair. The reduction in amplitude is expressed as a ratio or difference and represents the degree of gating that occurs. Higher ratios or smaller difference scores reflect weaker gating and previous work has associated weaker gating with impaired cognitive functioning in clinical populations such as schizophrenia (Erwin et al., 1998) and autism (Buchwald et al., 1992).

Taken together, the objective of the current study was to examine the effects of hypoxia on visual and auditory sensory ERPs that are both known to be affected by changes in the central nervous system. While previous work has shown that later, cognitive potentials are disrupted by hypoxia, here we hypothesized that prior to cognitive processing, sensory transduction is also impaired under acute hypoxic conditions. Based on previous literature in the visual and auditory domains, respectively, we predicted that the visual P100 component would be reduced in amplitude and that sensory gating, as measured with the auditory P50, N100, and P200 components would be impaired under hypoxic compared to normoxic conditions.

## Materials and Methods

### Participants

A total of 34 healthy adults (age: *M* = 29.12, *SD* = 6.19; 16 males) participated for monetary compensation. All participants were recruited through flyers and online announcements. Participants who completed the study received $150. The study protocol was approved by the Naval Medical Research Unit—Dayton’s (NAMRU-D) Institutional Review Board in compliance with all applicable federal regulations governing the protection of human participants. All participants self-reported normal or corrected-to-normal vision, normal hearing, no history of psychological, neurological, or medical diagnosis, no use of tobacco in the past 6 months, and no excessive alcohol use. Twenty-six participants self-reported previous experience being hypoxic prior to the current study. Of those 26 participants who reported previous hypoxia exposure, 19 reported prior exposure as part of a research study and 7 reported going through hypobaric chamber training with the U.S. Air Force.

### Procedures

**FIGURE 1:**
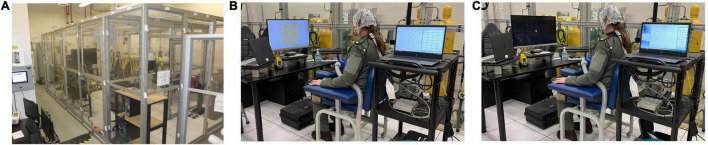
**(A)** Reduced Oxygen Breathing Environment (ROBE) where all study visits took place. **(B)** Visual experimental setup. **(C)** Auditory experimental setup. *Photos courtesy of NAMRU-D.*

Participants completed three study visits on separate days (exceptions noted below). All study visits took place in the Reduced Oxygen Breathing Environment (ROBE; Hypoxico, Inc.) at NAMRU-D. The ROBE is a normobaric hypoxia chamber (Figure 1A). The ROBE works by drawing in ambient room air and separating the oxygen molecules from the nitrogen molecules via a zeolite molecular sieve bed. The trapped, unwanted oxygen is exhausted while the hypoxic air, with a specific oxygen content, is pumped into the enclosure. This ultimately results in an environment that contains more nitrogen than normal ambient air, but there is no existing literature to suggest that a change from approx. 80% nitrogen (20% oxygen) to approx. 90% nitrogen (10% oxygen) would have any confounding effects on performance, physiology, and/or neural function. During the initial baseline visit, participants were presented with the visual and auditory paradigms (detailed below) separately in counterbalanced order. The baseline visit occurred under normoxic conditions (21% O_2_). During the second and third visits, the hypoxia visits, participants were presented with the visual paradigm in one visit and the auditory paradigm in the other visit with the order of these visits counterbalanced. For hypoxia visits, participants were exposed to a 9.7% O_2_ concentration for 14.5 min.

### Physiological Monitoring

During all study visits, both peripheral oxygen saturation (SpO_2_) and heart rate (HR) were monitored and recorded at a sampling rate of 1 Hz. Both measures were acquired via a Nonin finger-mounted pulse oximeter (Nonin Medical Inc.) and recorded by an iPad via Bluetooth connection. A safety cut-off criterion of 55% SpO_2_ was used.

### Hypoxia Symptom Questionnaire

The Hypoxia Symptom Questionnaire (HSQ) is a 15-item list describing typical hypoxia-related symptoms that was developed based on symptoms described in the didactic portion of Navy hypoxia familiarization training (Sausen et al., 2001). Participants were asked to rate each symptom on a 4-point scale (0 = not observed, 1 = mild, 2 = moderate, or 3 = severe). The symptoms included were: tingling, hot flashes, cold flashes, dizziness, tunnel vision, loss of consciousness, light dimming, euphoria, loss of coordination, headache, fatigue, breathlessness, blurred vision, nausea, and apprehension. Participants completed the HSQ after all three experimental visits: Normoxia, hypoxia for the visual paradigm, and hypoxia for the auditory paradigm.

### Electroencephalography Data Acquisition and Analysis

EEG data were recorded continuously from 32 electrodes covering the whole scalp with approximately uniform density using an elastic electrode cap (ActiCHamp, Brain Products) referenced to the right mastoid (TP10) in DC mode, at a sampling rate of 1,000 Hz. Electrode impedance for all channels was kept below 10 kΩ. EEG data were processed using the Fieldtrip software package (Oostenveld et al., 2011). Data were segmented into epochs covering the time from 100 ms before to 400 ms after the onset of each stimulus presentation. Additional EEG processing steps are detailed below for each experimental paradigm separately.

## Experiment 1: Visual

### Stimuli

Participants were seated approximately 90 cm from a 28 in diagonal monitor (Figure 1B). Stimuli were controlled by MATLAB (The MathWorks, Natick, MA) with Psychophysics Toolbox extensions (Brainard, 1997; Pelli, 1997). A pattern-reversal paradigm was used to elicit the visual P100 component (Halliday et al., 1973; Celesia and Brigell, 1999). A black and white checkerboard pattern was presented in the center of the screen. The total checkerboard subtended 19.5° of visual angle, and each individual check subtended 0.62°. The checks reversed without a change in total luminance. The pattern reversed at a rate of 1 Hz for a 30 s duration “trial.” A 5 s inter-trial interval was used. Participants underwent a total of 24 trials, which yielded 720 stimulus presentations total.

### Visual P100

After trial epochs were created, data were high-pass filtered at 1 Hz and low-pass filtered at 20 Hz, and re-referenced to Fz. Independent components analysis (ICA) was performed on epoched data and the eye blink component was removed for every participant. After ICA, EEG waveforms from frontal electrodes (i.e., Fp1, Fp2) were visually inspected to identify voltage fluctuations typical of gross motor movements (amplitude > 50 μV). Trials containing these types of artifacts were rejected entirely. After artifact rejection, average waveforms were calculated for an *a priori* group of occipital electrodes (Oz, O1, O2). For each dataset, the P100 was defined as the most positive going waveform between 50 and 110 ms. To compromise between peak- and mean-based measures, as described in Kappenman and Luck (2016), we reported the mean amplitude in a 20 ms window centered around the peak, such that the window varied for each dataset.

### Results

**FIGURE 2:**
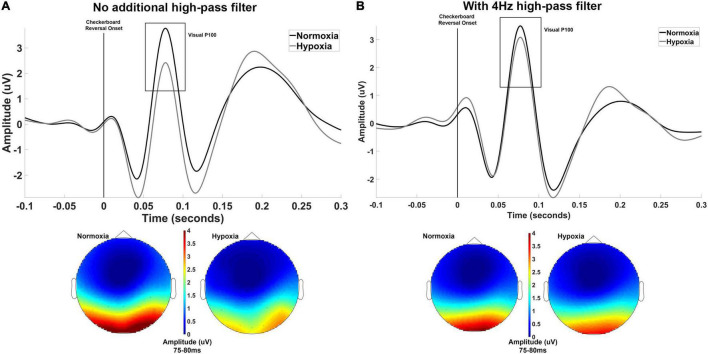
**(A)** Grand averaged waveforms averaged across electrodes Oz, O1, and O2 shown separately for normoxia and hypoxia with no additional high-pass filter. Also shown are scalp maps illustrating grand averaged amplitude from 75 to 80 ms for normoxia and hypoxia separately. With no additional high-pass filter, these results indicate a significant reduction in the P100 amplitude under hypoxic compared to normoxic conditions. **(B)** The exact same data are shown with the inclusion of a 4 Hz high-pass filter to account for slow shifts in the EEG signal. This additional filter yielded a non-significant difference in P100 amplitude between hypoxia and normoxia conditions.

Three participants did not complete the hypoxia visit for the visual experiment and are not included in the below analyses. The primary dependent variable of interest for the visual experiment was the mean amplitude of the visual P100 component. For one participant, no discernible P100 could be identified for the normoxia visit and this participant was excluded from further analyses, leaving a final sample of *n* = 30 for analysis. A paired-samples *t*-test was used to assess potential differences in the P100 amplitude between normoxia and hypoxia visits. The mean amplitude of the P100 was significantly reduced under hypoxic compared to normoxic conditions, *t*(29) = 4.322, *p* < 0.001, *d* = 0.79 (Figure 2A). A paired-samples *t*-test was also tested on P100 peak latency, but no significant difference between normoxia and hypoxia emerged, *t*(29) = –1.006, *p* = 0.323.

Upon examination of our grand average waveforms in Figure 2A, it appears that the reduction in amplitude of the P100 during hypoxia may have been influenced by the preceding negative peak’s amplitude. As discussed in Kappenman and Luck (2012), the amplitude of a deflection can be distorted by slow shifts, meaning an underlying component can lead to incorrect amplitude assessments of another component. To examine the possibility of this in our current data, we imposed a 4 Hz high-pass filter prior to calculating peak and mean amplitude to reduce the influence of the slow shifts that might distort our data. In doing so, we retested the above *t*-test on mean amplitude centered around the peak and found that the difference between hypoxia and normoxia conditions did not reach significance, *t*(29) = 1.862, *p* = 0.07, *d* = 0.34. The grand average waveforms with the 4 Hz filter applied are shown in Figure 2B.

## Experiment 2: Auditory

### Stimuli

To measure sensory gating, we used a paired-clicks, condition-test paradigm (Wood and Wolpaw, 1982). Auditory stimuli were presented to participants via Etymotic ER3-A insert earphones (Figure 1C). Participants were presented with identical pairs of 1 ms 1,000 Hz sinusoidal tones (1 ms rise/fall; 70 dB). The interval between the first (S1) and second (S2) click was 500 ms. The inter-pair interval was randomly selected between 6 and 10 s at 1 s intervals. A total of 105 pairs were presented. Participants fixated on a cross presented in the center of a 28 in monitor throughout the auditory paradigm.

### Auditory P50, N100, and P200

After trial epochs were created, data were high-pass filtered at 1 Hz and low-pass filtered at 50 Hz, and re-referenced to the average of both mastoids (TP9, TP10). ICA was performed on epoched data and the eye blink component was removed for every participant. After ICA, EEG waveforms from frontal electrodes (i.e., Fp1, Fp2) were visually inspected to identify voltage fluctuations typical of gross motor movements (amplitude > 50 μV). Trials containing these types of artifacts were rejected entirely. Prior to averaging, signals were filtered with a 10 Hz high-pass filter to optimize scoring of the P50 or with a 20 Hz low-pass filter to optimize scoring of the N100 and P200 (Jerger et al., 1992; Lijffijt et al., 2009). Average waveforms were then calculated at electrode Cz separately for S1 and S2. To quantify P50, N100, and P200 components, we followed the methods detailed in Lijffijt et al. (2009). First, we identified the N100 as the most negative going waveform between 50 and 150 ms with a fronto-central topography. The N100 had to be greater in amplitude than the noise (i.e., baseline period). Next, we identified the P50 as the most positive going waveform between 35 and 85 ms or the most positive going waveform preceding the N100. Finally, the P200 was identified as the most positive going waveform following the N100 and/or between 150 and 250 ms. Both gating ratios (S1/S2) and difference scores (S2–S1) have been used in the literature, but difference scores have been found to have higher test-retest reliability compared to ratios for the P50 (Fuerst et al., 2007). Therefore a stimulus (S1, S2) × condition (normoxia, hypoxia) repeated-measures ANOVA was used to test for differences by condition in what amounts to the difference score.

### Results

One participant did not complete the hypoxia visit for the auditory paradigm and is not included in the below analyses, leaving a final sample of *n* = 33 for analysis. Following previous literature, the primary dependent variables of interest for the auditory experiment were the peak amplitude of the P50, N100, and P200 components. For each component, we tested a 2 (stimulus: S1, S2) × 2 (condition: normoxia, hypoxia) repeated-measures ANOVA on peak amplitude.

**FIGURE 3:**
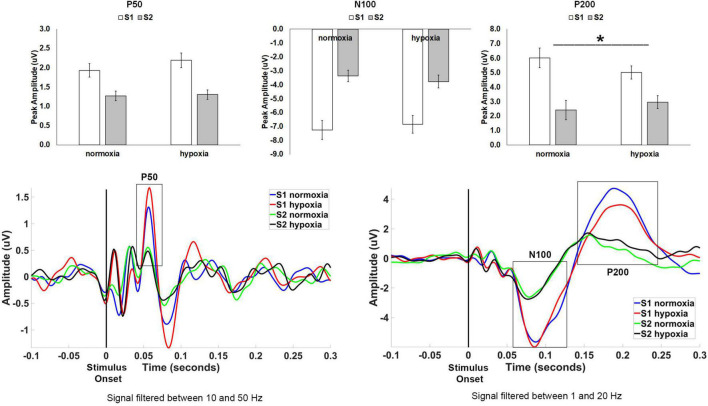
Top row: Average peak amplitude for the auditory paradigm, shown separately for the P50, N100, and P200 components. A sensory gating effect (i.e., reduced amplitude for S2 compared to S1) emerged for all three components. Only the P200 component showed a significant effect of hypoxia, with decreased sensory gating under hypoxic compared to normoxic conditions. *p < 0.05. Error bars represent standard error of the mean. Bottom row: Grand average waveforms at electrode Cz.

For P50 amplitude (Figure 3), the main effect of stimulus was significant as expected, *F*(1, 32) = 24.512, *p* < 0.001, η*_*p*_*^2^ = 0.434, with a larger amplitude for S1 compared to S2 indicating sensory gating. However, neither the main effect of condition, *F*(1, 32) = 2.332, *p* = 0.137, nor the stimulus × condition interaction, *F*(1, 32) = 1.731, *p* = 0.198, reached significance.

For N100 amplitude (Figure 3), the main effect of stimulus was significant as expected, *F*(1, 32) = 71.682, *p* < 0.001, η*_*p*_^2^* = 0.691, with a larger amplitude for S1 compared to S2 again indicating sensory gating. Neither the main effect of condition interaction, *F*(1, 32) < 0.001, *p* = 0.992, nor the stimulus × condition interaction, *F*(1, 32) = 2.719, *p* = 0.109, reached significance.

For P200 amplitude (Figure 3), the main effect of stimulus was again significant as expected, *F*(1, 32) = 28.624, *p* < 0.001, η*_*p*_^2^* = 0.472, with a larger amplitude for S1 compared to S2. The main effect of condition, *F*(1, 32) = 0.381, *p* = 0.542, was not significant. However, the stimulus × condition interaction, *F*(1, 32) = 7.482, *p* = 0.01, η*_*p*_^2^* = 0.190, was significant demonstrating a diminished sensory gating effect (i.e., difference between S1 and S2) for hypoxia as compared to normoxia.

## Cross-Experiment Exploratory Analyses

We examined the physiological and symptoms data for the visual and auditory experiments together for two reasons. First, the hypoxia exposures were identical, other than the stimuli presented, and we had no expectation that the stimuli presented would differentially affect SpO_2_, HR, and/or symptom presentation. Second, the repeated-measures nature of the design allowed for an opportunity to explore reliability of physiological response and symptom presentation across two identical hypoxia exposures. There is much anecdotal evidence in the aviation training community that inter-individual variability in response to hypoxia is high, but that intra-individual variability is low. In other words, individuals are thought to have a hypoxia “signature” that is consistent across exposures and this is the basis of hypoxia familiarization training for military aircrew (Neuhaus and Hinkelbein, 2014). However, the formal scientific evidence for this effect is limited (Smith, 2008; Tu et al., 2020).

### Physiological Measures

**FIGURE 4:**
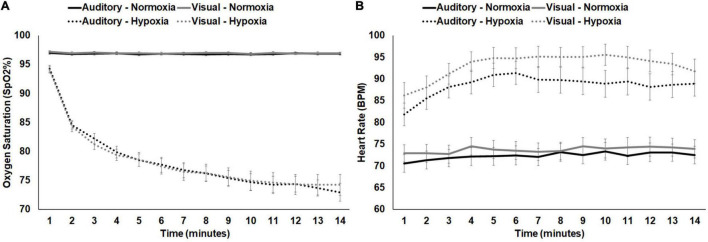
**(A)** SpO_2_ and **(B)** HR shown separately for normoxia and hypoxia and by experimental stimuli presented. Error bars represent standard error of the mean.

For average SpO_2_, a 2 (stimulus: visual, auditory) × 2 (condition: normoxia, hypoxia) repeated-measures ANOVA was tested. A main effect of condition emerged, *F*(1, 28) = 313.119, *p* < 0.001, η*_*p*_^2^* = 0.918, with significantly lower SpO_2_ during hypoxia compared to normoxia. Neither the main effect of stimulus, *F*(1, 28) = 0.034, *p* = 0.854, nor the stimulus × condition interaction, *F*(1, 28) = 0.044, *p* = 0.836, approached significance. For average HR, the same 2 × 2 ANOVA was tested. A main effect of condition emerged, *F*(1, 28) = 221.460, *p* < 0.001, η*_*p*_^2^* = 0.888, with significantly higher HR during hypoxia compared to normoxia. The main effect of stimulus was also significant, *F*(1, 28) = 6.190, *p* = 0.019, with higher HR during the visual stimuli compared to the auditory. However, the stimulus × condition interaction was not significant, *F*(1, 28) = 1.319, *p* = 0.261. Figures 4A,B shows average SpO_2_ and HR for each condition and stimulus separately, at 1 min intervals throughout the exposure. Additionally, correlation analyses demonstrated consistency across the two hypoxia exposures. Average SpO_2_ was significantly positively correlated for the visual and auditory paradigm exposures, *R*(29) = 0.694, *p* < 0.001. Similarly, average HR was also significantly correlated, *R*(29) = 0.616, *p* < 0.001. These results suggest that physiologically, participants responded similarly across two exposures to the same altitude on separate days.

### Hypoxia Symptoms

For self-report of hypoxia symptoms, a total HSQ score was calculated for each participant for each of the three visits (normoxia, visual hypoxia, and auditory hypoxia). A repeated-measures ANOVA was tested on visit with those three levels and showed a significant main effect of visit, *F*(1.531, 44.395) = 44.313, *p* < 0.001, η*_*p*_^2^* = 0.604. Bonferroni corrected *post hoc* comparisons showed that participants had higher HSQ scores for both hypoxia visits (visual: *M* = 7.27 ± 4.785; auditory: *M* = 7.03 ± 4.612) compared to normoxia (*M* = 1.27 ± 1.856), both *p*s < 0.001, but that there was no difference in scores when comparing visual and auditory hypoxia visits, *p* = 1.000.

**FIGURE 5:**
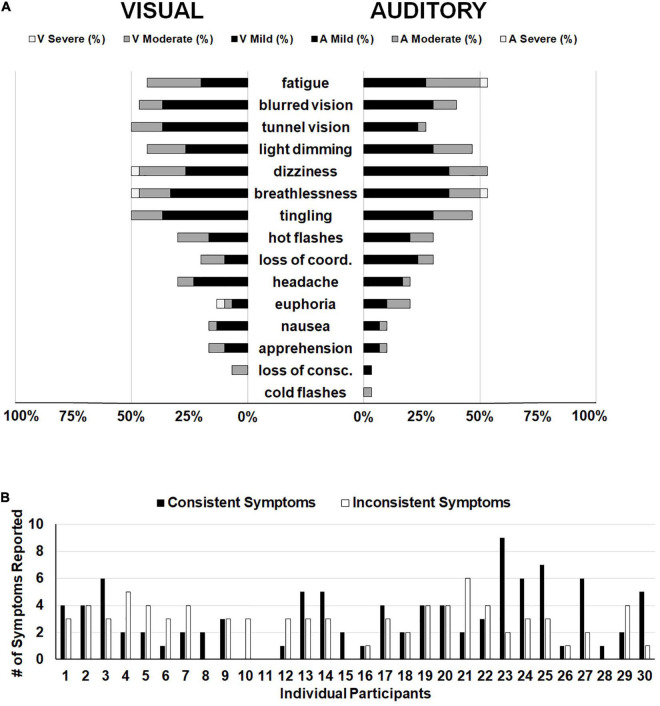
**(A)** Frequency of reporting for each item on the HSQ. The left side displays symptom reporting for the visual stimuli hypoxia session and the right side displays the auditory stimuli hypoxia session. Bars represent the distribution of severity reported for each symptom (i.e., black for mild, dark gray for moderate, and white for severe). Empty space reaching out to 100% represents the percentage of participants who did not report observing that particular symptom. **(B)** Individual participant data demonstrating the number of symptoms reported consistently (i.e., both hypoxia sessions; black bars) and the number of symptoms reported inconsistently (i.e., one hypoxia session, but not the other; white bars).

In order to focus on symptoms that were only attributable to hypoxia and not to extraneous variables such as the stimuli presented or time on task, we subtracted symptom ratings from the normoxia visit for the two hypoxia visits separately. In other words, if a participant reported mild fatigue (score of 1) during the normoxia visit and mild fatigue (score of 1) during the visual hypoxia visit, the resulting score was a zero (i.e., symptom not observed) for visual hypoxia because that fatigue cannot be attributed to the effects of hypoxia. Alternatively, if a participant reported mild blurred vision (score of 1) for normoxia and moderate blurred vision (score of 2) for hypoxia, then their hypoxia symptom rating for that visit would be a 1, because they experienced greater blurred vision with the addition of hypoxia. Using this approach, Figure 5A provides a visualization of symptom frequency and severity for each hypoxia visit.

The above reported ANOVA results suggest that as a group, our participants reported consistent symptoms across the two hypoxia exposures, as evidenced by a non-significant difference in the group mean total HSQ score between exposures. This approach of comparing group-level symptom presentation across multiple exposures predominates the existing literature on the topic (Woodrow et al., 2011; Johnston et al., 2012; Tu et al., 2020). However, it remains unclear whether individual participants experienced consistent symptom presentation and severity across the two exposures in the current study. To explore this question, we calculated the number of symptoms that each participant reported in both hypoxia visits (i.e., consistent symptoms) and we calculated the number of symptoms that each participant reported in only one of the hypoxia visits (i.e., inconsistent symptoms). Frequency of consistent and inconsistent symptoms can be seen in Figure 5B. If we define the presence of a “hypoxia signature” as individuals who had a higher number of consistent symptoms compared to inconsistent symptoms, then in our sample of 30 who completed both hypoxia visits, a total of 13 individuals showed a signature. This data suggests that the presence of a hypoxia signature may not be as common as traditionally thought (see section “Discussion” for additional considerations).

## Discussion

In the current study, we examined the effects of acute hypoxia exposure on early sensory evoked potentials in both the visual and auditory domains. Using a pattern-reversal paradigm, we found that the visual P100 was reduced in amplitude during hypoxia compared to a normoxia baseline visit. However, additional inspection of the waveforms and further filtering caused this effect to become non-significant. Secondly, using a test-condition auditory paradigm, we investigated the effects of hypoxia on sensory gating. Our results demonstrated that the earliest sensory gating ERPs, the P50 and N100, were not altered under hypoxic conditions, but the later P200 component was disrupted during hypoxia. Specifically, we found that P200 sensory gating was diminished during hypoxic compared to normoxic conditions. Finally, given the repeated-measures nature of our design, we engaged in some exploratory analyses to examine the consistency with which individuals reported subjective symptoms of hypoxia across identical exposures on separate days.

The effects of hypoxia on a number of visual performance measures have been well documented, such as color vision (Connolly et al., 2008; Barbur and Connolly, 2011), light perception (Fowler et al., 1993), and visual acuity (Leber et al., 1986). As noted by Connolly et al. (2008), the effects of hypoxia on the visual system have larger implications for human performance, such as contributing to slowed reaction times (RT). The literature on the negative influence of hypoxia on RT performance is also substantial and consistent (Fowler et al., 1982; Dart et al., 2017; Blacker and McHail, 2021). This relationship between effects on the visual system and effects on RT are difficult to disentangle with psychophysical studies alone. One advantage to the approach used in the current study is that the visual P100 is a passively elicited measure that is independent of any motor response by the participant. Thus, the current results suggest that visual impairment is not occurring at the level of visual cortex during hypoxia. Future work should examine other potential sources of this impairment, such as in the thalamus or even at the retina. One interesting caveat to the visual P100 results here is the effect of a stringent high-pass filter to control for slow shifts in the EEG signal. Both results are reported and illustrated to highlight the importance of proper methods and interpretation of ERP data (Kappenman and Luck, 2012). The original data analysis showed a robust difference in amplitude between hypoxia and normoxia, but the high-pass filtered results moved this result to a *p*-value that did not reach significance (i.e., 0.07). Therefore, these results are interpreted cautiously and future work should aim to replicate (or not) the effect. Further, the role of low oxygen exposure in the slow changes in the EEG signal should be investigated. For example, are those changes truly artifact unrelated to hypoxia or could they be representative of some meaningful neural marker of hypoxia that could be further explored?

The current study showed that the early sensory gating components, the P50 and N100, were unaffected, but the later P200 showed a significant reduction in sensory gating. Previous work suggests that the early P50 reflects a different sensory gating mechanism compared to the N100 and P200 components (Boutros et al., 2004). Further, it has been suggested that the N100 gating represents a filtering mechanism involved in triggering attention, whereas the P200 gating relates to filter mechanisms involved in allocation of attention (Lijffijt et al., 2009). This implication of impaired allocation of auditory attention during hypoxia exposure is in line with previous work using an auditory oddball paradigm demonstrating reduced amplitude MMN (Blacker and McHail, 2021) and P3a (Seech et al., 2020) components. The MMN/P3a response complex is thought to track the detection and orienting of attention to a deviant stimulus (Polich and Criado, 2006; Näätänen et al., 2007). Given the results of the current and previous studies, it seems that unlike the visual system, auditory information processing is only affected at the level of attention and not at a sensory processing level during acute hypoxia exposure. However, the effects of hypoxia on attentional allocation to auditory stimuli have important consequences for aviator performance, namely communication between aircrew and between aircrew and ground crew, as well as in-cockpit spatial audio cueing systems.

Military aviation training involves deliberate hypoxia exposure in a controlled environment known as hypoxia familiarization training (Artino et al., 2006). The goal of this training is to allow trainees to experience hypoxia and identify their symptoms, as well as practice emergency procedures during a low-oxygen exposure. The premise is that knowing one’s individual symptom profile will allow for faster identification of a hypoxic exposure in the aircraft if an incident were to occur. However, this training approach relies on the assumption that individuals are consistent in their hypoxia symptom profile (i.e., a hypoxia “signature”) from day to day and across years between trainings. To date, there are few studies that have attempted to examine the consistency of hypoxia symptoms across exposures. Those that have, have primarily relied upon recollections of hypoxia symptoms that occurred years prior for one of the two comparison exposures (Woodrow et al., 2011; Johnston et al., 2012; Tu et al., 2020). The current study is unique in that both symptom questionnaires were administered immediately following an actual hypoxia exposure. Previous studies have also been largely anecdotal and non-experimental, relying on descriptive statistics for the basis of their interpretations. When statistical tests were used, either to compare mean scores or to determine the degree of association between classifications, these analyses were all conducted at the group level. These types of analyses can be problematic because they are ill-equipped to answer the question of whether a hypoxia symptom “signature” exists. If two groups appear to be statistically similar, that doesn’t necessarily dictate that intra-individual reliability is high. The current study attempted to provide a novel approach to examining the degree of symptom congruence on the individual level. Although outside the scope of this paper, future research should attempt to investigate better ways to measure the phenomenon of a hypoxia symptom signature.

While the current study helps narrow the search of where and when hypoxia impacts sensory processing, limitations of the study merit consideration. First, circumstances vary widely in mishaps involving hypoxia, including the duration, and altitude of the exposure. While the current study assessed the impacts of hypoxia at a single altitude, to determine the generalizability of these findings and whether a dose-response relationship exists for the severity of hypoxia, future studies might compare the effects of multiple altitudes on sensory evoked potentials. To aid in transitioning these findings to the aviation training environment, sensory evoked potentials could also be monitored in individuals undergoing a standard hypoxia profile used in aviation survival training. Additionally, the majority of the participants in the current study reported prior experience with hypoxia exposure or hypoxia training. While it is unlikely that neural indices of pre-attentive sensory processing assessed in this study would be influenced by experience, future efforts might control for hypoxia training or directly compare responses in populations with or without hypoxia familiarization training.

The current study assessed sensory evoked potentials in the visual and auditory systems and found that neural markers of attentional auditory function were disrupted by hypoxia, but early markers of visual processing were not significantly disrupted. These findings have important implications for hypoxia familiarization training in aviation safety programs and may lead to novel assessment tools that can be used in future investigations of the effects of hypoxia on the nervous system.

## Data Availability Statement

The raw data supporting the conclusions of this article will be made available by the authors, without undue reservation.

## Ethics Statement

The studies involving human participants were reviewed and approved by the Naval Medical Research Unit—Dayton’s IRB. The patients/participants provided their written informed consent to participate in this study.

## Author Contributions

KB generated the idea for the study, preprocessed the EEG data, performed the statistical analyses on all data, and wrote the first draft of the manuscript. DM critically edited the first draft of the manuscript. KB and DM designed the experiment and collected the data. Both authors approved the final submitted version of the manuscript.

## Author Disclaimer

The views expressed in this article reflect the results of research conducted by the authors and do not necessarily reflect the official policy or position of the Department of the Navy, Department of Defense, nor the U.S. Government.

## Conflict of Interest

The authors declare that the research was conducted in the absence of any commercial or financial relationships that could be construed as a potential conflict of interest.

## Publisher’s Note

All claims expressed in this article are solely those of the authors and do not necessarily represent those of their affiliated organizations, or those of the publisher, the editors and the reviewers. Any product that may be evaluated in this article, or claim that may be made by its manufacturer, is not guaranteed or endorsed by the publisher.
